# Fuzzy Controller Applied to a Remote Energy Harvesting Emulation Platform

**DOI:** 10.3390/s20205874

**Published:** 2020-10-17

**Authors:** Marcelo Miranda Camboim, Juan Moises Maurício Villanueva, Cleonilson Protasio de Souza

**Affiliations:** Renewable and Alternatives Energies Center (CEAR)/Electrical Engineering Department (DEE)/Campus I, Federal University of Paraiba (UFPB), Joao Pessoa 5045, Brazil; jmauricio@cear.ufpb.br (J.M.M.V.); protasio@cear.ufpb.br (C.P.d.S.)

**Keywords:** fuzzy controller, energy harvesting, thermal emulation

## Abstract

In the last decades, a lot of effort has been made in order to improve the use of environmentally friendly and renewable energy sources. In a context of small energy usage, energy harvesting takes place and thermal energy sources are one of its main energy sources because there are several unused heat sources available in the environment that may be used as renewable energy sources. To rapidly evaluate the energy potential of such thermal sources is a hard task, therefore, a way to perform this is welcome. In this work, a thermal pattern emulation system to evaluate potential thermal source in a easy way is proposed. The main characteristics of the proposed system is that it is online and remote, that is, while the thermal-source-under-test is being measured, the system is emulating it and evaluating the generated energy remotely. The main contribution of this work was to replace the conventional Proportional Integral Derivative (PID) controller to a Fuzzy-Proportional Integral (PI) controller. In order to compare both controllers, three tests were carried out, namely: (a) step response, (b) perturbation test, (c) thermal emulation of the thermal pattern obtained from a potential thermal source: tree trucks. Experimental results show that the Fuzzy-PI controller was faster than the PID, achieving a setting time 41.26% faster, and also was more efficient with a maximum error 53% smaller than the PID.

## 1. Introduction

Energy harvesting is defined as the practice of capturing energy available in the environment as, for instance, sunlight [1], heat [2], movement [3], and others, and converting it into electric energy to be stored or consumed [4]. A common usage for the harvested energy is to power low-power electronic devices as sensors and microcontroller-based boards and some applications are, for example, wireless sensors networks for environmental monitoring [5], aeronautics applications [6], and photovoltaic panels monitoring [7].

Thermal energy is one of most used sources in energy harvesting, because heat is generated by many applications and released in the environment [8]. Many thermal sources have already been exploited, for example, body heat [9], rooftops [10], and aircraft [11]. However, early evaluating the energy potential of a thermal source is a hard task if the source is located in a hard-to-access place.

In order to convert thermal energy into electric energy, generally, thermoelectric generators (TEGs) are used. A TEG is a device made of an array of *pn* semiconductor junctions connected electrically in series and thermally in parallel by two ceramic plates [12]. Thermal energy is converted into electric energy when a difference of temperature is on the TEG’s faces (the ceramic plates).

To early evaluate the energy-generating potential of thermal sources in a remote way, an emulation system capable of applying any thermal pattern onto a TEG and verifying the generated voltage was proposed in [13]. The main contribution of this system is the online emulation, that is, while the difference of temperature is measured in the remote energy source to be tested, this difference of temperature is being emulated in a thermal platform in a laboratory located far away from the thermal source.

In [13], a conventional Proportional-Integral-Derivative (PID) controller was projected to control a thermal emulation platform. Although more than 90% of the controllers used in the industrial sector are PID [14], this type of controller is not suitable for non-linear and time variant systems [15,16], as an example the proposed platform [17]. In this way, many research opportunities have taken place, such as: intelligent controllers [18], adaptive [19], hybrids [20], etc. However, although these controllers can be implemented in other ways, implementations based on Fuzzy Logic are more effective, because control strategies lies on the designer experience [21] instead of mathematical models. In addition, Fuzzy-based controllers are implemented with many inputs activating the outputs only when certain conditions are established, making Fuzzy Logic computationally efficient in the control of systems that presents non-linear and time variant behavior [22,23].

The main goal of this work is the development of a Fuzzy-PI system to control the thermal emulation platform described in [13] in order to emulate remotely and optimally the thermal patterns obtained from an interesting thermal source, tree trunks [5].

The main obtained results of this work are: (a) The development of a Fuzzy-PI controller to the platform capable of stabilizing the temperature onto TEG faces with a maximum steady state error equal to 0.091 ${}^{\circ }$C (53% less when compared with the steady state error obtained from the PID controller); (b) achievement of robustness to external disturbances and plant parameters variation; and (c) the capacity to emulate thermal patterns using an intelligent controller based on Fuzzy Logic.

This work is structured as follows: in Section 2, the online thermal emulation system responsible for measuring the temperatures of a thermal source and emulating remotely are discussed. In Section 3, the Fuzzy controller implementation is discussed, from the definition of input variables to the definitions of the rules. In Section 4, validation tests of the proposed Fuzzy controller, and results analysis are shown. Finally, the final considerations are presented in Section 5.

## 2. Proposed System

**Figure 1:**
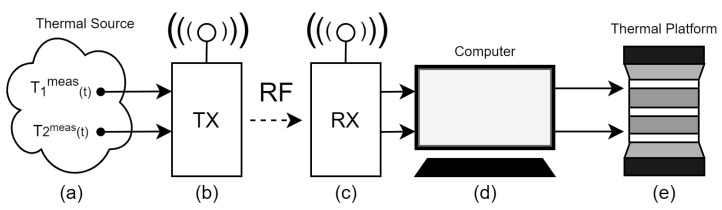
Proposed online emulation system. (**a**) Thermal source and the measurement device. (**b**) Transmitter. (**c**) Receiver. (**d**) Computer. (**e**) Thermal emulation platform.

**Figure 2:**
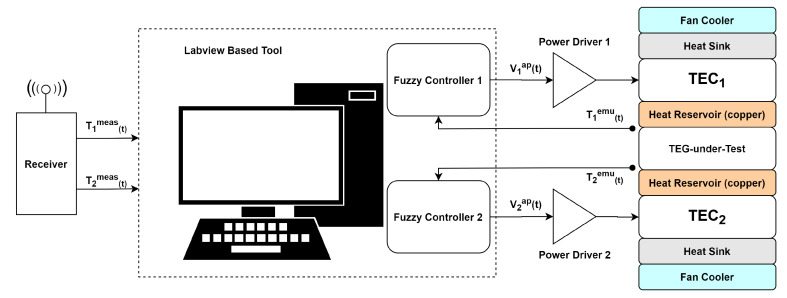
Thermal emulation system block diagram.

In [13], a remote thermal emulation platform was presented that allows to acquire temperature values from a remote thermal source and, at the same time, to emulate these in laboratory. Then, the platform is very useful to early validate remotely the energy potential of hard-to-access sources. In Figure 1, the platform diagram is shown. Its main components are:

1 Two temperature sensors to measure the remote thermal source: $T_{1}^{\mathrm{meas}}$(t) and $T_{2}^{\mathrm{meas}}$(t);
2 TX: RF radio transmitter;
3 RX: RF radio receiver;
4 Computer: run the Fuzzy controllers, using $T_{1}^{\mathrm{meas}}$(t) and $T_{2}^{\mathrm{meas}}$(t) as their set-points, and generate the actuating signals $V_{1}^{\mathrm{ap}}$(t) and $V_{2}^{\mathrm{ap}}$(t); and
5 Thermal platform: receive the $V_{1}^{\mathrm{ap}}$(t) and $V_{2}^{\mathrm{ap}}$(t) and, after passing through power drivers, apply them to the upper cooler (TEC${}_{1}$) and the bottom cooler (TEC${}_{2}$), respectively, resulting in the actual temperature, T${}_{1}^{\mathrm{emu}}$(t) and T${}_{2}^{\mathrm{emu}}$(t), that are applied onto the TEG-under-test faces. The TEG-under-test generates an electric voltage proportional to the temperature difference ($\Delta T\;=\;T_{1}^{\mathrm{emu}}\left(t\right)-T_{2}^{\mathrm{emu}}\left(t)\right)$, as shown in the right side of the Figure 2.

### 2.1. Emulation Platform

**Figure 3:**
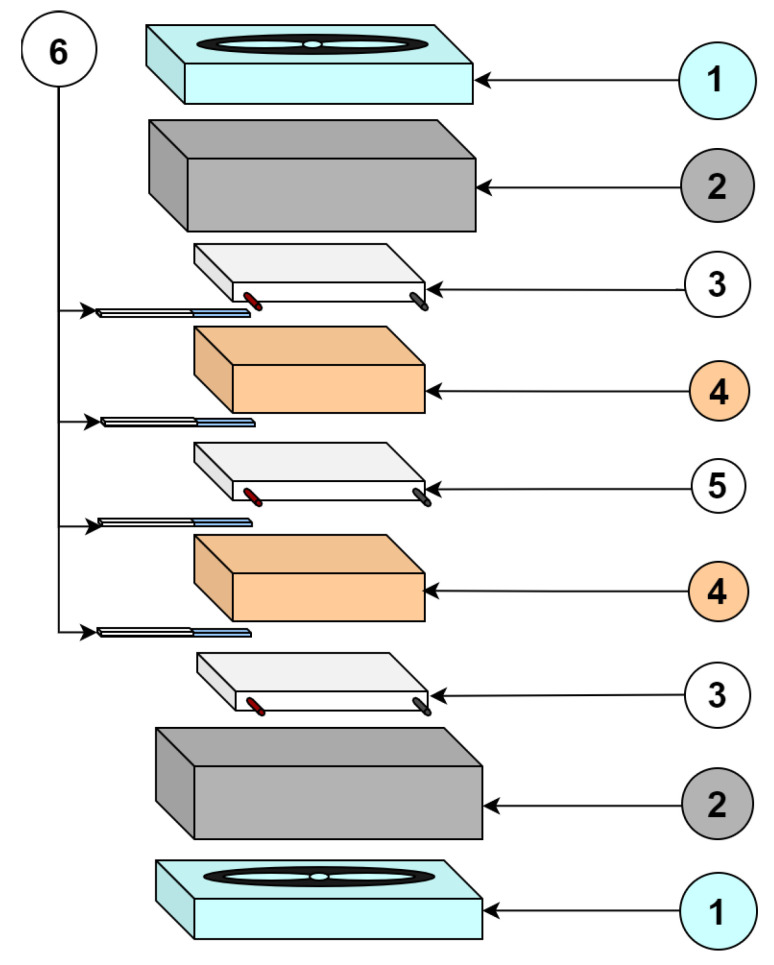
Emulation platform. (1) Fan coolers, (2) heat sinks, (3) thermoelectric coolers (TECs) as coolers, (4) copper blocks, (5) thermoelectric generator (TEG), and (6) thermocouples. Source: ©2020 IEEE. Reprinted, with permission, from IEEE Transactions on Instrumentation and Measurement [13].

The emulation platform was firstly proposed in [24], the diagram and the physical structure of the platform is shown in Figure 2 and Figure 3, respectively. This platform features a symmetrical structure composed by two fans, two modules (TEC${}_{1}$ and TEC${}_{2}$), acting as thermoelectric coolers (TEC), and a module acting as a thermoelectric generator (the TEG-under-test or just TEG). Between each thermoelectric pair (TEC${}_{1}$/TEG and TEG/TEC${}_{2}$), there is a copper plate acting as a thermal reservoir, which conducts heat from/to a TEC to/from the TEG. The thermal reservoir works as thermal damp easing the control action. To measure temperature, 4 thermocouples have been used and they are placed on the surfaces of the upper and bottom faces of the thermal reservoirs.

The main proposal of this work was to improve the control of the variables T${}_{1}^{\mathrm{emu}}$(t) and T${}_{2}^{\mathrm{emu}}$(t) using two independent Fuzzy-PI controllers, one for each variable. As described earlier, the controller’s set-points are $T_{1}^{\mathrm{meas}}$(t) and $T_{2}^{\mathrm{meas}}$(t), and the proposed controllers act on the actuating voltage signal applied into the coolers TEC${}_{1}$ and TEC${}_{2}$, respectively, $V_{1}^{\mathrm{ap}}$(t) and $V_{2}^{\mathrm{ap}}$(t). The control system procedure was developed using LabView™ and the Data Acquisition (DAQ) Board PCIe-6321, by National Instruments.

### 2.2. Fuzzy Controller

As discussed in [17], Peltier cells suffer physical degradation in their life time. In this way, the parameters of a TEG, for instance, internal resistance, Seebeck coefficient, and thermal conductivity, vary along the time.

(1) $$u\left(t\right)=K_{p}e\left(t\right)+K_{i}\int_{0}^{t}e\left(t\right)dt+\frac{de(t)}{dt}$$

As a consequence, if a conventional PID controller is used to control the temperature levels on the TEG’s faces, the well known PID’s parameters $K_{p}$, $K_{i}$ and $K_{d}$, shown in Equation (1) have to be periodically updated. Other observed issues in using a conventional PID controller is the presence of noise during the thermal emulation due to the ambient temperature variation and of electric noise due to the temperatures measurements.

Considering this scenario, a Fuzzy-PI controller is proposed to control T${}_{1}^{\mathrm{emu}}$(t) and T${}_{2}^{\mathrm{emu}}$(t). The controller has two input variables, namely, the temperature error, $E_{i(t)}=T_{i}^{\mathrm{meas}}\left(t\right)-T_{i}^{\mathrm{emu}}\left(t\right)$ and the error temperature variation, $\Delta E_{i(t)}=E_{i(t)}-E_{i(t}-T)$, where i =1, 2 (1 for the first Fuzzy controller and 2 for the second one) and T is the sampling period.

The Fuzzy controller output in an instant t is given by Equation (2), providing an action of integration to the system.

(2) $$V_{i}^{\mathrm{ap}}\left(t\right)=V_{i}^{\mathrm{ap}}(t-T)+\Delta V_{i}^{\mathrm{ap}}\left(t\right)$$

## 3. Controller Implementation

In the project of a Fuzzy controller there are plenty of choices and options, different from conventional controllers. The configuration considering two inputs (error and error variation) showed up to be more frequently used, providing good results in many applications as, speed control to synchronous motors [25] and DC motor [26], gas turbine [27], electromagnetic launchers [28], hydraulic excavator [29], and others. Table 1 summarizes the studies on Fuzzy-PI controllers cited above.

**Figure 4:**
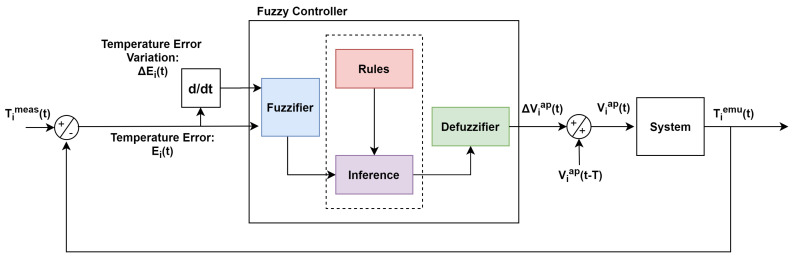
Fuzzy Logic diagram.

On the other hand, the controller performance depends on adjustments that can be made by trial and error, or even using evolutionary strategies using genetic algorithm, for example. The main advantages of a Fuzzy controller is that: (1) The designer does not need to know the exact model of the system. (2) The Fuzzy controller is able to handle possible variations in the system. (3) The rules can be established using natural language based on the designer’s experience [25]. In this section, the steps taken in order to implement the Fuzzy-PI temperature controller for the emulation platform are described. The steps were taken considering the Fuzzy diagram shown in Figure 4.

### 3.1. Range of the Variables

The first step to implement the proposed Fuzzy controller is the fuzzification of the input variables. As the chosen main application of the described thermal emulation platform is to harvest energy from tree trunks (*Smart Tree*) [5], two reference temperatures were considered, namely, the internal tree temperature in a depth of 100 mm and the external ambient temperature. In this case, two temperature sensors were installed into a tree trunk at the Federal University of Paraiba (UFPB), located in the city of João Pessoa, Brazil, a city near to the equator line.

The ranges of the controller variables are: (a) Temperature: it was considered that the temperatures can vary from 20 ${}^{\circ }$C ($T_{min}\left(t\right)$) to 40 ${}^{\circ }$C ($T_{max}\left(t\right)$); (b) temperature error: the range of the temperatures error was considered varying from −20 ${}^{\circ }$C ($T_{min}\left(t\right)-T_{max}\left(t\right)$) to 20 ${}^{\circ }$C ($T_{max}\left(t\right)-T_{min}\left(t\right)$); (c) temperature error variation: from the system behavior, it was verified that the maximum temperature error variation is 0.3 ${}^{\circ }$C; (d) applied voltage variation: the output voltage can assume values from −10 V to 10 V, however, in order to have softer and more stable variations, the range of −3 V to 3 V was chosen. Table 2 shows these values.

### 3.2. Fuzzification

As the system is complex and non linear, the input and output variables were grouped in seven different categories in order to make the system more robust, namely: Positive big (PB), medium (PM), positive small (PS); zero (Z); negative big (NB), negative medium (NM), and negative small (NS). The numerical limits to each category were defined considering the previous knowledge about the platform behavior, and the chosen membership function was the triangular one, since it is one of the most popular [30] mainly because of its advantage of simplicity using straight lines.

Specifically, the sets according to the defined categories are:

- Temperature:
  1 Between −20 ${}^{\circ }$C and −7 ${}^{\circ }$C. The error is considered NB because it was observed that, in this range, the voltage applied into the TECs can be a maximum without causing an overshoot in the temperature during the transient response.
  2 Between −11 ${}^{\circ }$C and −3 ${}^{\circ }$C. The error is considered NM because, in this range, the system is near to the steady state value and there is no need to apply high voltages that can destabilize the system.
  3 Between −6 ${}^{\circ }$C and 0 ${}^{\circ }$C. The error is considered NS because, in this range, the system is very near to the steady state, demanding only small adjustments.
  4 Between −1 ${}^{\circ }$C and 1 ${}^{\circ }$C. The error is considered Z, with only a few adjustments necessary to maintain the temperature in the current value.
  5 The same logic is applied into the positive groups: PS, PM, and PB, but with positive signal values.
- Temperature error variation:
  1 Between −0.3 ${}^{\circ }$C and −0.15 ${}^{\circ }$C, and 0.15 ${}^{\circ }$C and 0.3 ${}^{\circ }$C, the variation was considered as NB and PB, respectively, because these variations occur only when a high voltage is applied.
  2 In the other sets, the range between −0.225 ${}^{\circ }$C and 0.225 ${}^{\circ }$C was equally divided.
- Voltage variation: the range was equally divided, meaning that:
  1 Between −3 V and −2 V, NB.
  2 Between −3 V and −1 V, NM.
  3 Between −2 V and 0 V, NS.
  4 Between −1 V and 1 V, Z.
  5 The same for the positive values.

**Figure 5:**
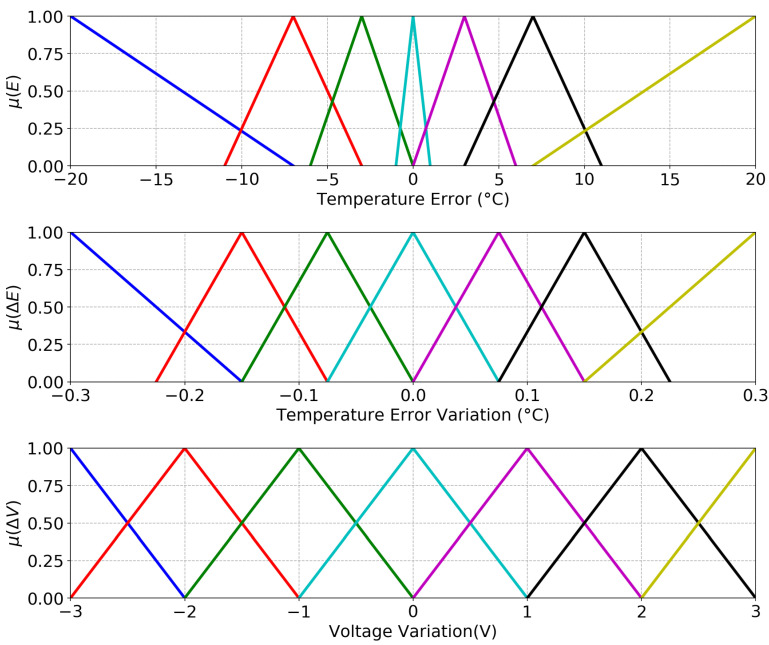
Membership functions. Negative big (blue line), medium (red line), small (green line), zero (cyan line), positive small (purple line), medium (Black), big (Yellow).

The fuzzified variables are shown in Figure 5.

### 3.3. Rules Determination

**Figure 6:**
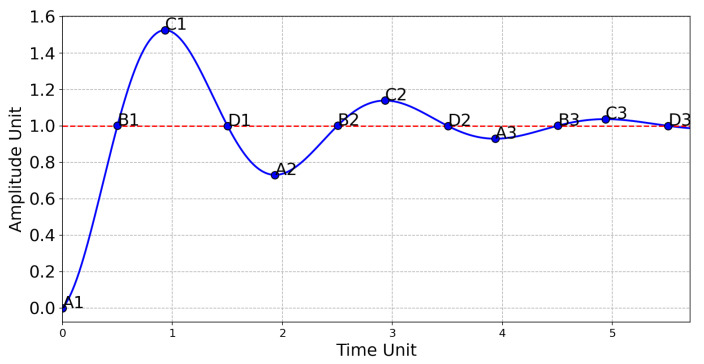
Graph used to define the rules of the system.

In order to define the Fuzzy rules, the response of an undamped system was considered. Figure 6 shows some particular cases of situations that can occur in the system.

The rules were extracted considering the points shown in Figure 6 and are the following: A1: the error is positive and big (PB) and the variation is null (Z), so the voltage variation should be positive and big (PB); B1: the error is null (Z) and the variation is positive and big (PB), so the voltage variation should be negative and big (NB) to decrease the variation; C1: the error is negative and big (NB) and the variation is null (Z), so the voltage should be negative and big (NB); D1: The error is null (Z) and the variation is negative and big (NB), so the voltage variation should be positive and big. For the the remaining points, the logic was the same, obtaining the rules described in the Table 3.

### 3.4. Defuzzification

**Figure 7:**
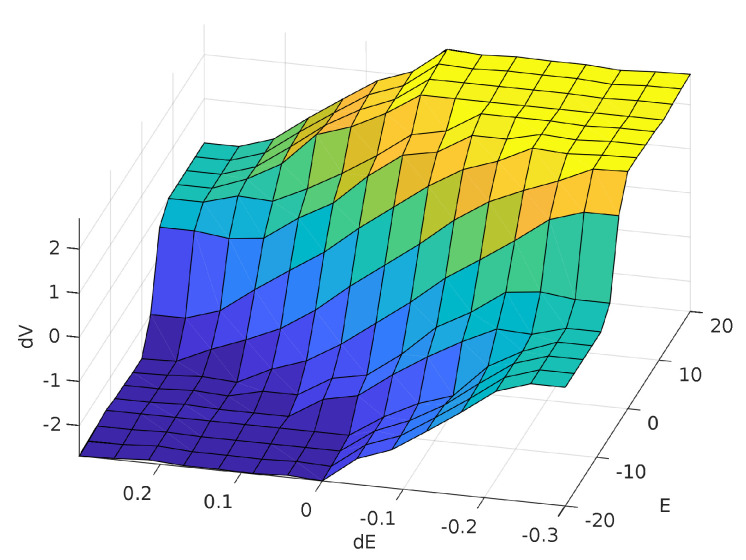
Relationship between the inputs: Error variation (dE) and error (*E*); and the output: voltage variation (dV).

**Figure 8:**
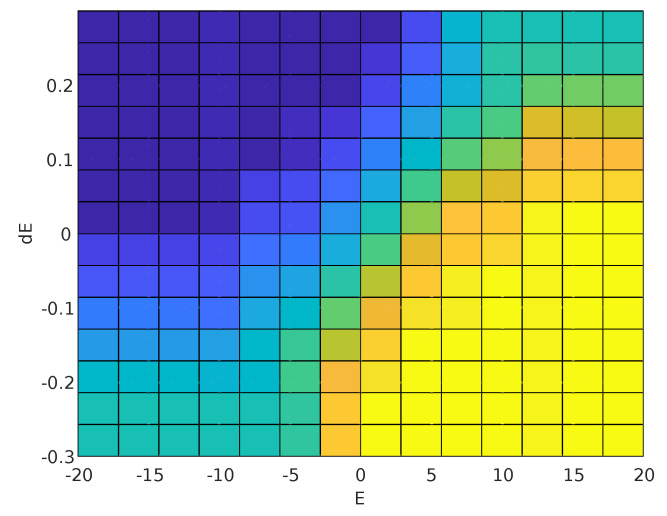
Upper view of the relationship between the inputs: Error variation (dE) and error (*E*); and the output: voltage variation (dV).

From the rules, the defuzzification process was done using the Center of Area method. Figure 7 shows a surface with error and error variation as the inputs and the voltage variation as the output ($\Delta V_{i}^{ap}\left(t\right)=f\left(E_{i}\left(t\right),\Delta E_{i}\left(t\right)\right)$), and Figure 8 shows an upper view of the surface.

In Figure 7, the surface can be divided in sub-regions, namely: (a) **Saturation region**: the surface has two regions of saturation (negative saturation in blue and positive saturation in yellow) with values equal to ±3V. The output of the system reaches this values when the error or/and the voltage variation are far away from zero; (b) **Steady region**: this region is indicated in light blue and represents the state where the voltage variation is almost null. This occurs when the error is very small or when the error is relatively high, but the error variation is already big enough; and (c) **Convergence region**: this region indicates the other cases, when the system is operating in the intermediate regions in order to reach the surface center.

## 4. Results

Using the thermal emulation platform, 3 distinct experiments were carried out in order to compare the efficiency of the proposed Fuzzy controller with a conventional PID controller. The gains of the PID controller were obtained using Ziegler–Nichols method followed by small adjustments, the optimum parameters were obtained comparing the steps responses of the adjusted gains. The experiments are: (a) Step response: temperature steps were applied into the system and the temporal response was verified; (b) perturbation: an external perturbation was applied into the system and the response of the controller was verified; (c) online emulation experiment: the thermal platform was used to emulate a thermal pattern in an remote way, the thermal patterns are composed by two temperatures, the internal temperature of a tree trunk in a depth of 100 mm and the external temperature.

### 4.1. Step Response

**Figure 9:**
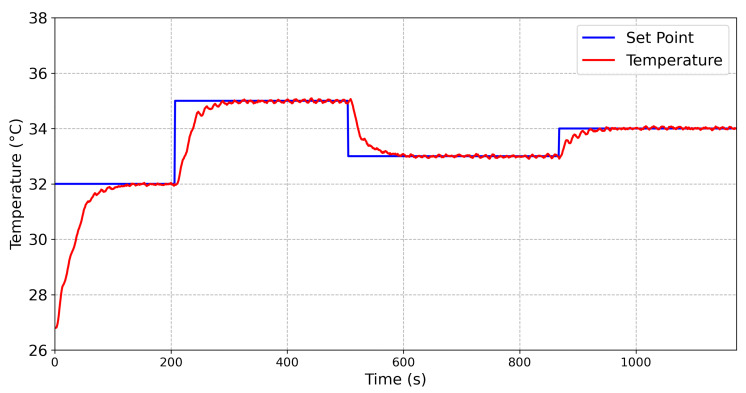
Relationship between the set-point (blue line) and the emulated temperature (red line) for the step response of the proposed Fuzzy controller.

**Figure 10:**
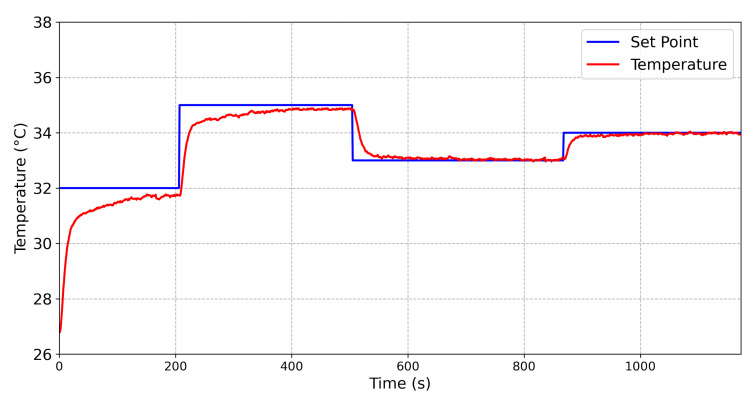
Relationship between the set-point (blue line) and the emulated temperature (red line) for the step response of a PID controller.

To compare the behavior of the controllers, temperature steps were applied into one face of the TEG, while the temperature in the other face was constant. Then, the main parameters of a step response were obtained from both controllers. The results obtained from the Fuzzy controller and the conventional PID controller are shown in Figure 9 and Figure 10, respectively.

The results obtained from a temperature variation from 32 ${}^{\circ }$C to 35 ${}^{\circ }$C are described in Table 4. It can be seen that, for the Fuzzy controller, a settling time ($T_{s}$) equal to 78 s was obtained, while the $T_{s}$ for the PID controller was 189 s. The maximum steady state error was 0.091 ${}^{\circ }$C for the Fuzzy controller, while the maximum steady state error for the PID controller was 0.17 ${}^{\circ }$C. Thus, the Fuzzy controller was faster and more accurate than the conventional PID controller.

### 4.2. Perturbation Experiment

To evaluate the robustness of the Fuzzy controller against external perturbations, the following procedure was done for both controllers.

- After the system reached equilibrium at room temperature, a temperature set-point equal to 32 ${}^{\circ }$C was defined onto one face of the TEG;
- After the system reached the steady state, a cooler fan, placed very near to the platform, was turned on;
- By convection, the cooler fan introduces an external perturbation into the system.

**Figure 11:**
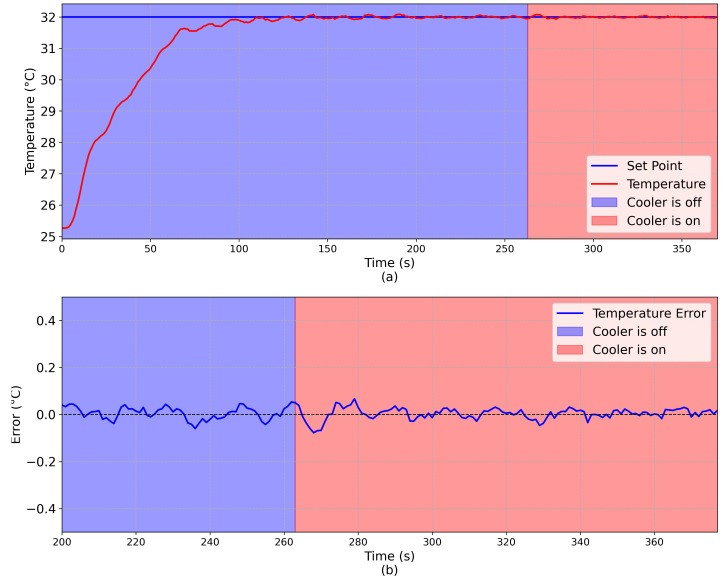
Fuzzy: (**a**) Comparison between the set-point (blue line) and the emulated temperature (red line). (**b**) Error (E = Set-Point - Emulated Temperature) during the noise test. The region with a blue background indicates that the cooler is off and the region with a red background indicates that the cooler is on.

**Figure 12:**
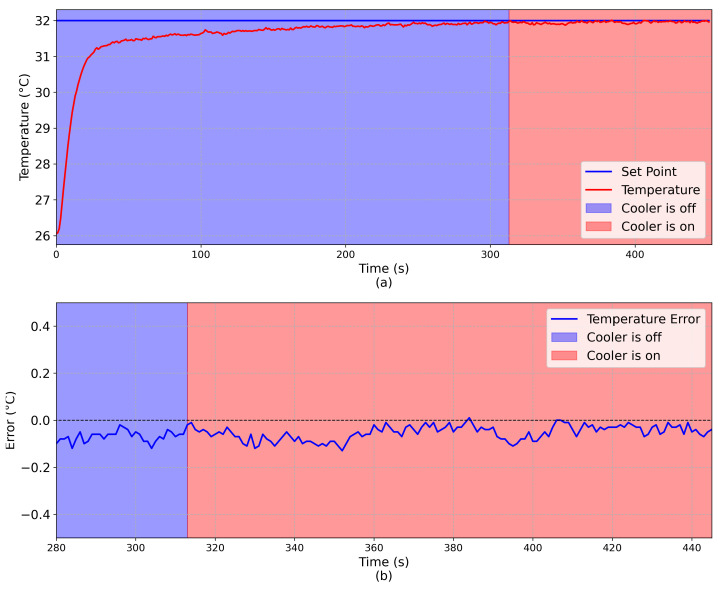
PID: (**a**) Comparison between the set-point (blue line) and the emulated temperature (red line). (**b**) Error (E = Set-Point - Emulated Temperature) during the noise test. The region with a blue background indicates that the cooler is off and the region with a red background indicates that the cooler is on.

The complete experiment is shown in Figure 11 and Figure 12, considering the Fuzzy and PID controllers, respectively. Considering the Fuzzy controller, it can be observed that, when the fan cooler is turned on at t = 263 s, there was a high temperature variation because of the convection. However, the projected Fuzzy controller was robust enough to compensate this perturbation. Considering the PID controller, the cooler was turned on at t = 313 s, and as it can be seen that the system takes almost 50 s to return to the steady state condition, proving that the Fuzzy controller is more suitable to deal with external perturbations.

### 4.3. Online Emulation

**Figure 13:**
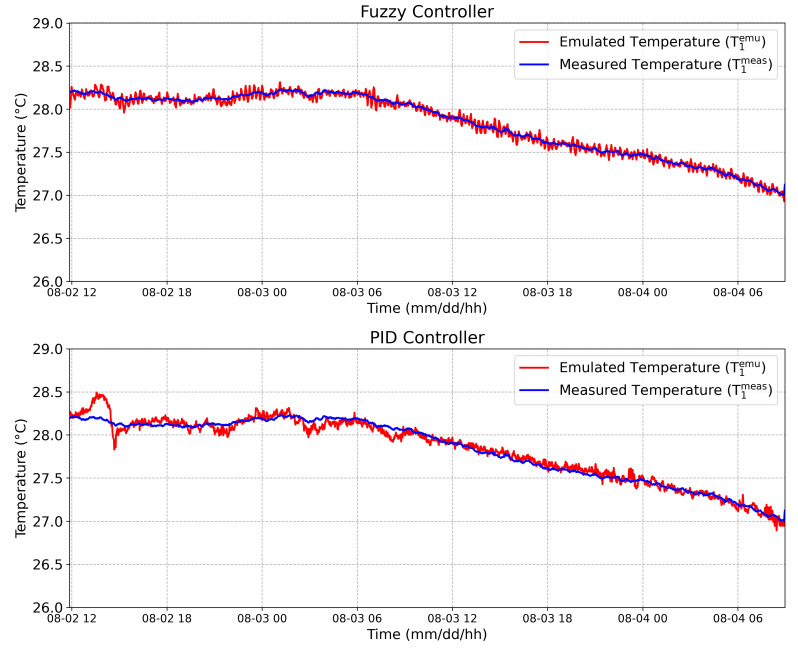
The 100 mm depth internal temperature: Measured temperature on the upper face $T_{1}^{\mathrm{meas}}$ (blue line), and the emulated temperature on the upper face, $T_{1}^{\mathrm{emu}}$ (red line), considering the Fuzzy controller and the PID controller, respectively.

**Figure 14:**
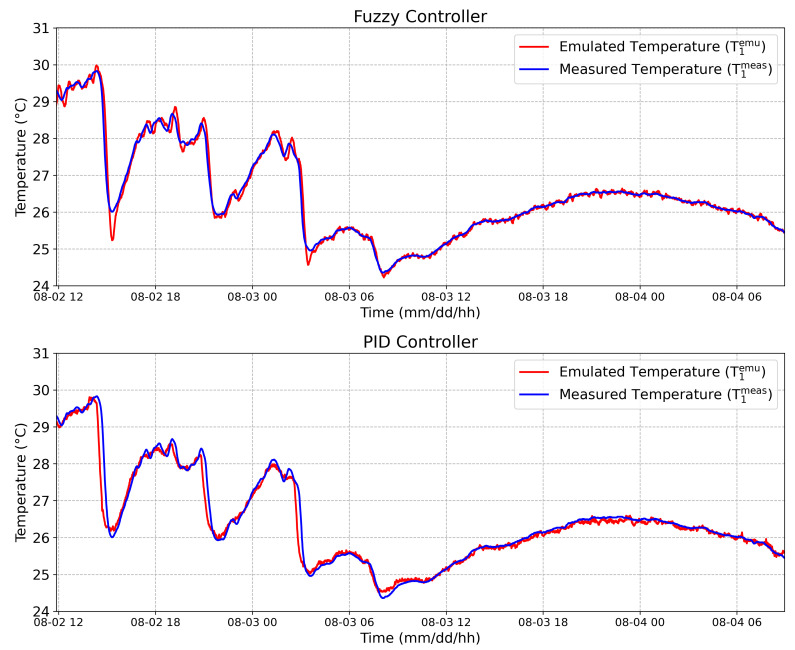
External temperature: Measured temperature on the bottom face $T_{2}^{\mathrm{meas}}$ (blue line), and the emulated temperature on the bottom face, $T_{2}^{\mathrm{emu}}$ (red line), considering the Fuzzy controller and the PID controller, respectively.

Using the complete system proposed in Section 2, a thermal pattern was obtained and emulated in an remote way using the PID controller as described in [13], then the same pattern was applied into the Fuzzy controller. This thermal pattern is composed of two temperature signals, namely: the internal temperature into a tree trunk at 100 mm depth ($T_{1}^{\mathrm{meas}}$(t)) and the external temperature ($T_{2}^{\mathrm{meas}}$(t)). After, this thermal pattern was emulated in an remote way, obtaining T${}_{1}^{\mathrm{emu}}$(t) and T${}_{2}^{\mathrm{emu}}$(t). The results are showed in Figure 13 and Figure 14.

In order to evaluate the controllers’ performance, the root mean square error (RMSE) was calculated using the Equation (3), where *N* is the total number of samples and *T* is the sampling time.

(3) $$RMSE_{i}=\sqrt{\frac{1}{N}\sum_{n=1}^{N}{\left(T_{i}^{meas}\left(nT\right)-T_{i}^{emu}\left(nT\right)\right)}^{2}}$$

Considering the internal emulated temperature (upper face), an RMSE equal to 0.04 ${}^{\circ }$C and 0.07 ${}^{\circ }$C were obtained by the Fuzzy controller and by the PID controller, respectively. Considering the external emulated temperature (bottom face), an RMSE equal to 0.15 ${}^{\circ }$C and 0.14 ${}^{\circ }$C were obtained by Fuzzy controller and by the PID controller, respectively. Although the RMSE considering the external temperature was 6.7% higher in the Fuzzy controller compared to the PID, the RMSE obtained from the Fuzzy controller was 57% smaller than the PID for the internal temperature emulation. This result is very reasonable considering that the temperature sensor responses are acquired with a sampling rate set up by a 12-bit ADC and transmitted by radio and, as a result, with the 12-bit ADC at 3.3 V reference voltage, the achieved overall resolution is 0.08 ${}^{\circ }$C [13].

**Figure 15:**
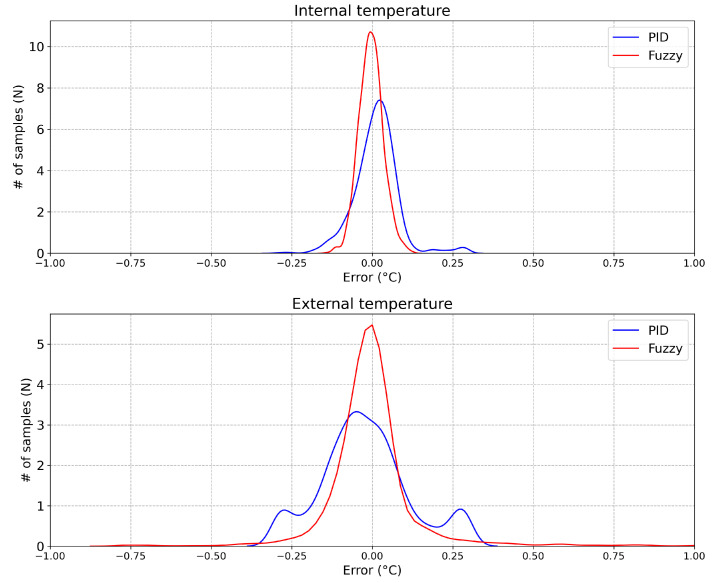
Error distribution of the PID controller (blue line) and Fuzzy controller (red line), considering the internal temperature errors and the external temperature errors, respectively.

Moreover, it can be seen in Figure 15 the error’s distribution, $E_{1,2}\left(t\right)=T_{1,2}^{meas}\left(t\right)-T_{1,2}^{emu}\left(t\right)$, related to the emulated temperatures in the Figure 13 and Figure 14, considering both controllers. In these histograms, all the 2523 samples were taken into consideration.

It can be seen that, for the internal temperature (upper face), the errors in both controllers are well distributed forming the shape of a Gaussian function. The mean value of the error distribution (μ) was −0.004 ${}^{\circ }$C by the Fuzzy controller and 0.011 ${}^{\circ }$C by the PID controller. Considering the standard deviation (σ), a value equal to 0.037 ${}^{\circ }$C was obtained by the Fuzzy controller and 0.069 ${}^{\circ }$C by the PID.

Considering the external temperature (bottom face), the error in the Fuzzy controller was well distributed forming the shape of a Gaussian function, but this was not true when the PID controller was taken into consideration. The mean value of the Fuzzy controller’s error distribution (μ) was almost 0.000 ${}^{\circ }$C and 0.011 ${}^{\circ }$C by the PID controller. Thus, a standard deviation (σ) equal to 0.151 ${}^{\circ }$C was obtained by the Fuzzy controller and 0.138 ${}^{\circ }$C by the PID.

The results are summarized in Table 5 and Table 6. As it can be seen, the performance of the Fuzzy controller in the online emulation is better than the PID.

## 5. Conclusions

In this work, Fuzzy controllers were proposed in order to control two distinct temperature levels in a system capable of emulating thermal patterns in a remote way. The obtained results show that the Fuzzy controller achieves a steady state error 53% smaller and a settling time 41.26% smaller when compared with a conventional PID controller. In addition, the proposed Fuzzy controller was robust enough to correct variations caused by external factors as, for example, variations at room temperature and convection. Considering thermal emulation, it was observed that the changes in the system dynamics introduced by the controller made the emulation more reliable, with an RMSE equal to 0.04 ${}^{\circ }$C and 0.15 ${}^{\circ }$C for the upper face and bottom face, respectively. Therefore, the Fuzzy controller shows up to be more suitable to the proposed application. As future work, the Fuzzy controller rules will be extracted automatically using neuro-fuzzy techniques, because one of the main limitations of the proposed approach is that the rules extraction depends exclusively on the designer’s knowledge.

## Figures and Tables

**Table 1:** Summary of previous studies related to Fuzzy-PI controllers.

Author	Proposition	Results
Sant et al. [25]	Control the speed of a PERMANENT MAGNET synchronous motor using a hybrid PI, that is, the output can be given by the conventional PI controller, by the Fuzzy controller or by an weight sum of both.	The hybrid Fuzzy controller performing better than the Fuzzy controller alone and the PI alone, reducing the start current in 23% when compared with the PI speed controller.
Khanke et al. [26]	Compare a PI controller, a Fuzzy controller and a hybrid Fuzzy PI controller performance in order to control the speed of a commuted brushless DC motor.	It was obtained that the Fuzzy logic controller and the Fuzzy-PI controller worked better than the PI controller, presenting lower settling times, rising times and peak overshot.
Kim et al. [27]	Compare the performance of a conventional PI controller with a Fuzzy-PI controller in a application that requires the control of the temperature and speed of a heavy-duty gas-turbine.	The overshoot of the rotor speed and exhausted temperature for the proposed Fuzzy-PI controller decreased considerably when compared with the conventional PI controller.
Li-Yi et al. [28]	Control the mover velocity of a electromagnetic launch, controlling the magnetic traveling field of a permanent magnetic linear synchronous motor using a Fuzzy-PI controller.	The system was able to adjust the mover velocity, with a reaction quicker and with a smaller overshoot.
Li et al. [29]	Control a hydraulic excavator using a Fuzzy-PI controller.	The Fuzzy-PI controller was able to overcome nonlinearity, parameter uncertainties and external disturbances, exhibiting a good performance when compared with a conventional PID controller and a Fuzzy Logic controller.

**Table 2:** Ranges related to each variable.

Variable	Range
Temperature (${}^{\circ }$C)	20 → 40
Temperature Error (${}^{\circ }$C)	−20 → 20
Temperature Error Variation (${}^{\circ }$C)	−0.3 → 0.3
Applied Voltage Variation (V)	−3 → 3

**Table 3:** Rules of the System.

E\Δ E	NB	NM	NS	Z	PM	PB	PS
NB	Z	NS	NM	NB	NB	NB	NB
NM	Z	Z	NS	NM	NB	NB	NB
NS	PM	PS	Z	NS	NM	NB	NB
Z	PB	PM	PS	Z	NS	NM	NB
PS	PB	PB	PM	PS	Z	NS	NM
PM	PB	PB	PB	PM	PS	Z	Z
PB	PB	PB	PB	PB	PM	PS	Z

**Table 4:** Step response parameters for each controller.

Variable	Fuzzy	PID
Settling Time ($T_{s}$)	78 s	189 s
Raise Time ($T_{r}$)	43 s	114 s
Peak Time ($T_{p}$)	119 s	202 s
Dead Time ($T_{d}$)	5 s	4 s
Maximum Error	0.091 ${}^{\circ }$C	0.17 ${}^{\circ }$C

**Table 5:** Results for the internal temperature emulation.

Variable	Fuzzy	PID
RMSE	0.040 ${}^{\circ }$C	0.070 ${}^{\circ }$C
Mean	−0.004 ${}^{\circ }$C	0.011 ${}^{\circ }$C
Standard deviation	0.037 ${}^{\circ }$C	0.069 ${}^{\circ }$C
Gaussian Shape	Yes	Yes

**Table 6:** Results for the external temperature emulation.

Variable	Fuzzy	PID
RMSE	0.150 ${}^{\circ }$C	0.140 ${}^{\circ }$C
Mean	0.000 ${}^{\circ }$C	0.011 ${}^{\circ }$C
Standard deviation	0.151 ${}^{\circ }$C	0.138 ${}^{\circ }$C
Gaussian Shape	Yes	No

## Abbreviations

The following abbreviations are used in this manuscript:

ADC	Analog to digital converter
DAQ	Data Acquisition
$E_{i}$	Error between the emulated temperature and the set-point temperature considering the i-th controller
NS	Negative small
NM	Negative medium
NB	Negative big
PB	Positive big
PID	Proportional-Integral Derivative
PM	Positive medium
PS	Positive small
RMSE	Root mean squared error
$T_{i}^{meas}$	Measured Temperature in the i-th sensor
$T_{i}^{emu}$	Emulated Temperature by i-th controller
$T_{m}ax$	Maximum Temperature
$T_{m}in$	Minimum Temperature
TEG	Thermoelectric Generator
TEC	Thermoelectric Cooler
$V_{i}^{ap}$	Applied voltage by the i-th controller
Z	Zero
μ	Mean value
σ	Standard deviation
